# The promise of long-term effectiveness of school-based smoking prevention programs: a critical review of reviews

**DOI:** 10.1186/1617-9625-5-7

**Published:** 2009-03-26

**Authors:** Brian R Flay

**Affiliations:** 1 Department of Public Health, Oregon State University, Corvallis, Oregon, USA

## Abstract

I provide a review and critique of meta-analyses and systematic reviews of school-based smoking prevention programs that focus on long-term effects. Several of these reviews conclude that the effects of school-based smoking prevention programs are small and find no evidence that they have significant long-term effects. I find that these reviews all have methodological problems limiting their conclusions. These include severe limiting of the studies included because of performance bias, student attrition, non-reporting of ICCs, inappropriate classification of intervention approach, and inclusion of programs that had no short-term effects. The more-inclusive meta-analyses suggest that school-based smoking prevention programs can have significant and practical effects in both the short- and the long-term. Findings suggest that school-based smoking prevention programs can have significant long-term effects if they: 1) are interactive social influences or social skills programs; that 2) involve 15 or more sessions, including some up to at least ninth grade; that 3) produce substantial short-term effects. The effects do decay over time if the interventions are stopped or withdrawn, but this is true of any kind of intervention.

## Background

Researchers and others have developed many school-based tobacco prevention programs over the past 30 years. Early approaches to smoking prevention went through several phases: informational, affective/motivational and psychosocial. Thompson [1], in a review of all English language papers on smoking prevention between 1960 and 1976, concluded that most methods evaluated up to that time, i.e., informational and affective approaches, were not effective and this was echoed by Beattie [2]. Many programs can change knowledge, but such change is not, by itself, enough to alter behavior [3] and, in any case, quickly decays [4]. Sometimes, information can actually make behavior worse [3,5] as can some affective programs [6]. U.S. Government agencies concluded during the late 1980's and early 1990's that traditional approaches (informational and affective) were largely ineffective and that the approaches based on social-psychological models [7,8] were modestly effective across a variety of settings, times and populations [9-12].

Over a dozen reviews of approaches to tobacco control or substance abuse prevention published since the early 1990's have included school-based smoking prevention within their realm [9,11,13-27]. Some of these reviews were broad-based and non-systematic, and some were very systematic. Earlier reviews of this type always included school-based smoking prevention as a critical component of effective broad-based tobacco control. Many of the later reviews, especially after Lantz et al [18] tended not to include school-based prevention as an important component in broad-based tobacco control. Lantz et al [18] concluded that "The long term impact of school based educational interventions is of concern" (page 49). However, they then emphasize the need to combine school-based prevention with media programming, other tobacco control efforts, and other problem behavior prevention efforts. Dobbins et al [28] recently concluded that "there is reason for optimism regarding the effectiveness of prevention programs on smoking behavior and initiation, albeit in the short term." (page. 296).

During this same period there were many reviews [10,28-37] and meta-analyses [4,34,38-43] of school-based smoking prevention. These reviews and meta-analyses repeatedly reinforced the fact that informational and affective programs do not work to change behavior. Furthermore, meta-analyses further (established the fact that some psychosocial programs and strategies, particularly those that are interactive programs based on the social influences approach (educating youth about social norms and influences and providing skills for resisting such influences), can be effective.

However, findings in the field are sometimes confusing to practitioners and policy makers because some early or short psychosocial programs reported promising short-term effects that did not last [44-48]. In addition, some tested programs simply were not effective [49]. DARE is a prime example of a program that seems to be similar to many successful programs in many ways, yet it has been proven in multiple studies and two meta-analyses [50-52]. These mixed results have led some to question the overall value of school-based smoking prevention [53].

I now provide a critical review of findings from prior meta-analyses and reviews. In an accompanying paper [54] I will provide a review of selected school-based smoking prevention programs with the promise of long-term effectiveness.

## Review of meta-analyses and the cochrane review

My objective is to provide a critical review of past reviews to determine whether or not school-based smoking prevention can be effective. Meta-analyses of school-based prevention programs have used various criteria and so have varied in scope, from focusing only on the quality of eleven evaluations (and not their outcomes) [34], to including 74 smoking prevention studies among 207 substance prevention studies [41], including evaluations of 65 separate programs [4], to reviewing 94 randomized trials of school-based smoking prevention but reviewing only 23 of then in detail because of methodological limitations with the rest [33]. Reviews of the long-term effects have also varied in scope from including 25 studies with at least 2-year follow-ups [21], to including only 8 studies with grade 12 (or age 18) outcome data [43]. The result has been a confusing array of findings, ranging from precise effect sizes for some types of programs [4,41] to a conclusion that most school-based prevention programs do not work [43,53]

Nan Tobler [55] summarized her series of meta-analyses and suggested that *programs that used interactive learning strategies and involved same- or similar-age peers as leaders or facilitators were most effective*. Tobler and colleagues found that smoking prevention programs produced an average effect size of 0.16, with "interactive" programs producing a significantly larger effect size than non-interactive programs (0.17 versus 0.05) [41]. Tobler and colleagues [41] also found that programs that address multiple substances were less effective at reducing tobacco use than programs that targeted only tobacco (ES = .10 vs. .17) – but they had the added benefit of reducing alcohol and other substance use as well. They also found program effects to be larger in schools with predominantly special or high-risk (minority, high absenteeism or dropout, poor academic records) populations. This is an important finding suggesting that these programs can reduce the gap between low- and high-risk groups of adolescents.

Hwang and colleagues [4] estimated an average short-term effect size of .19 for smoking behavior outcomes from the 65 programs they reviewed. They reported effect sizes of .22 for attitudes and skill, and .53 for knowledge. They found that all effects were smaller at delayed follow-ups. Behavior barely decayed over 1–3 years (to .18) but decayed by half (.09) at follow-ups of 3 or more years without further programming. Knowledge decayed dramatically by 1 year follow-up (to .19), and attitudes and skills decayed to about half their original effects by 1 year follow-up (.10 and .09, respectively).

Hwang and colleagues [4] also estimated the effects of different approaches to smoking prevention operationally defined as follows. Social Influence (SI) programs addressed immediate health, social, and cosmetic effects of smoking; peer and media influences; social norms, expectations, and acceptance; and other information; as well as social skills such as modeling, role-playing, and/or group practice. Cognitive-behavioral (CB) programs included the elements of the SI approach plus at least two cognitive skills such as problem-solving, decision-making, assertiveness, self-control, and/or other coping skills. Life Skills (LS) programs included the components of the SI and CB programs plus at least one affective skill such as self-confidence (self-efficacy), values clarification, and/or generic social skills. A second way of distinguishing programs was by their setting levels: exclusively school-based only or school plus community settings. The latter setting level was defined as including at least one of the following: community members (mass media, community key workers, parent/family members) and any community-involving activity such as homework assignments, awareness activities, organized campaigns, sports or cultural activities; efforts to develop and enforce a policy on tobacco use in schools or community; or volunteer work in the community.

Hwang et al. [4] found that social influences approaches had average effect sizes for short-term, 1–3 years and > 3 years smoking prevention, respectively, of .12, .15 and .07; cognitive behavioral approaches had effect sizes of .21, .21 and NA; Life skills of .29, .16 and NA. I do not consider these differences in effect sizes between types of programs to be very meaningful because a) it is very difficult to categorize programs accurately and, in any case, many of the differences between types of programs have, in practice, been minimized over time as researchers interacted and mixed components, and b) some of the differences between categories may be due to single research groups conducting multiple studies of one program that obtained unusually large effect sizes, as Hwang et al pointed out. For example, Steve Schinke and Lew Gilchrist (University of Washington) published results from 7 different small-scale studies of programs that Hwang categorized as using the CB approach, in which students rather than schools were randomly assigned to conditions, with higher effect sizes than most other programs and few long-term follow-ups. Similarly Gilbert Botvin conducted 9 of the 12 studies classified in this meta-analysis as life skills, with an average effect size of .44, considerably larger than the others in that category.

Regarding setting, Hwang et al [4] found that school-only programs reported effect sizes of .22, .16 and .06 at short-term, 1–3 years and > 3 years, respectively; and school plus community programs reported effect sizes of .16, .21, and NA. Again, I do not place much confidence in these estimates because a) the school plus community category includes such a wide range of different types of activities and b) the underlying school program varied greatly among them. In a previous systematic review of school and school plus community programs [56], I concluded that *school plus community programs produce about double the effect when the type of school program is held constant*.

Rooney and Murray's [40] meta-analysis of 131 smoking prevention programs adjusted for studies with a unit of analysis error, although this had little effect on the overall effect sizes. The average effect size was around 0.10 at long-term follow-up. This would approximate to a 4% relative reduction in smoking. Using a modeling approach, the authors estimated that the impact could be increased if programs began around sixth grade as part of a multi-component health program, gave same-age peer leaders a role in program delivery, and used booster sessions. They estimated that this might achieve a relative reduction in smoking of between 19% and 29%.

Thomas and Perera [33] completed the most thorough systematic (Cochrane) review of school-based smoking prevention studies to date with a minimum of 6 months follow-up after the completion of the intervention. They reviewed only randomized trials and found 94 of them (115 others identified as RTCs were eliminated because the reviewers determined that they were not really RCTs or because the follow-up was less than 6 months after the end of the intervention). They rated the methodological biases of each study and separated studies into those with minimal risk of bias (category 1), medium risk of bias (category 2) and high risk of bias (category 3). Six areas of possible bias were considered for their bias rating: 1) selection bias (baseline differences due to imperfect randomization or no report of exactly how randomization was conducted or whether it was concealed), 2) performance bias (due to problems with program implementation or contamination of the control group), 3) attrition bias (due to attrition rates of 20% or more or differential attrition by condition), 4) detection bias (due to differences in outcome assessment), 5) statistical power bias (due to inadequate power or no power analysis reported), and 6) statistical bias (due to inappropriate analysis such as not taking account of clustering or ICCs were not reported). Based on these ratings, they found and analyzed in detail only 23 studies of the highest quality (that suffered from the least bias). Of the remainder of the studies, 31 were rated to be in category 2 and 40 in category 3.

These are rather rigid criteria, and even many high-quality studies of school-based programs cannot meet them all, partly because many of the criteria are outside the control of researchers and partly because of editorial decisions by journals. For example, performance bias is likely in any school-based effectiveness trial, where regular school teachers or other providers deliver the program, compared with an efficacy trial, in which research staff deliver the intervention. However, this kind of bias is likely to lead to an underestimation rather than an overestimation of program efficacy, although potentially a true estimate of effectiveness under real-world conditions. The second kind of performance bias, contamination of the control group has become more likely historically, at least in most developed countries, as more and more schools already have some form of smoking prevention program, often derived from the same theoretical principles as the program being evaluated. Again, however, such bias is likely to lead to an underestimation of program effectiveness (because the control group is doing smoking education in their "business-as-usual" mode rather than no smoking education).

Attrition is an issue that was more of a problem when the field considered the student as the unit of analysis. Now that we (correctly) take the school to be the unit of analysis (as well as of random assignment and program delivery), the amount of attrition at the individual student level is less of an issue (as long as it is similar across conditions in both magnitude and type). In addition, attrition is likely to be high in schools with high turnover, the very schools many studies have deliberately selected for their studies because students in these schools are at highest risk for smoking. Non-differential attrition may reduce external validity (generalizability) of study results but not the internal validity (bias). Although attrition should be assessed and any differential attrition reported (and adjusted for in analyses if appropriate), *studies should not automatically be penalized based on levels of student attrition*. On the other hand, if one or more schools drops out of a study, that would be a great cause for concern.

Another consideration is that journals often will not publish all of the details of studies. For example, they often do not care to know whether randomization was concealed or not. They usually do not require that ICCs be reported, and only a few researchers have reported them. Analytical approaches have changed over time as statisticians have developed the methods to handle clustered data. Thomas and Perera determined statistical significance from their own analysis of odds ratios – the odds of baseline never smokers starting to smoke by posttest in the intervention group compared to the control group. When ICCs were not reported, they assumed an ICC of .097, the average found in a limited set of older studies [57]. This approach probably has lower statistical power that necessary and, in many cases, led to a decision that a difference that was reported as significant by the original authors was judged to be non-significant by these reviewers. Thus, this approach leads to a bias against finding significant effects.

Another criterion that might be overly strict is requiring at least one assessment at least 6 months beyond the end of the intervention. As interventions have become more comprehensive and longer-lasting, it is becoming more difficult to meet this standard. For example, many programs include some type of follow-up sessions during high school. It is not clear that a study should be excluded from consideration in a review because the last posttest was less than 6 months after the last session, albeit at the end of high school, when the bulk of the intervention may have occurred several years earlier.

### It is not clear that eliminating many studies because of the types of issues discussed above is beneficial to the field

It leads to the omission from consideration of many important studies, at least just as many of which may be biased to an underestimate of effect size as are biased to an overestimate of effect size. The overwhelming focus on methods is also at the expense of an informed focus on the interventions. A better approach would be thorough meta-analyses that provide analyses of the effects of methodological issues as well as programmatic ones [58].

Regarding program types, Thomas and Perera assigned studies to the groups of information-only, social competence (the Good Behavior Game and the Seattle Social Development Program), social influences (56 trials, 13 of which met the criteria for category 1), combined social competence/influences (e.g., Life Skills Training, Towards No Tobacco, Child Development Project), and multimodal (i.e., including family or community components). Some of the assignments are questionable. One glaring question, for example, concerns why most of Gilbert Botvin's studies of Life Skills Training were assigned to the combined social influences/competence category, yet one was assigned to the social influences group. A study by Ausems and colleagues [59] and another by Crone and colleagues [60] were of interventions that clearly included social influences components but they were categorized in the information-only category. There are many other examples of questionable assignment; as Thomas and Perera acknowledge (page 10), it is extraordinarily difficult for people not intimately involved in the field to determine how to group the different interventions. Most prior reviews and meta-analyses by people outside of prevention have had major problems with this. Nan Tobler probably did the best job of overcoming this, particularly in her later papers as she learned more and more about the programs she was dealing with. In addition, over time, the programs have become more and more alike as they incorporate ideas from each other. There is no longer much that separates some of the programs assigned to the social competence and social influence groups.

The only outcome reported by Thomas and Perera was the prevalence of smoking among pretest never smokers. They did not include other possible outcomes, such as changes in the proportion of ever, weekly or monthly smokers. This also unnecessarily limited the studies that were considered.

Within all of the above constraints, Thomas and Perera concluded that:

1. There is little evidence that information alone is effective (only one study in this category met their inclusion criteria).

2. Nine (which they usually characterize as half in their text) of 13 studies of social influences that met their criteria demonstrated effects and 4 did not.

3. The most rigorous and long-lasting test (65 lessons over 8 years) of a social influences program (Hutchinson) was not effective (see further discussion of this study below).

4. There was limited evidence for the effectiveness of social competence programs (only two studies met criteria for inclusion).

5. Of only 3 high-quality studies of the combination of social competence and social influences, only one showed a significant effect and one showed a significant effect only for the health-educator led condition (but not the "self-instruction" condition).

6. Three of the 4 studies of multimodal approaches that met standards for inclusion produced positive effects (characterized in the text as providing limited evidence of effectiveness).

7. There is little evidence of the long-term effectiveness of smoking prevention programs.

The conclusions reached in this review are overly restrictive for several reasons. First, as pointed out above, too many studies were eliminated from consideration for suspicions of bias that are unreasonably strict. Second, evaluations of DARE, a program that is known to be ineffective from several RCTs and two prior meta-analyses were included with the social influences group. Although DARE is partly based on the social influence approach, it is clearly a very poor example of it (see further discussion below). Third, the focus on the Hutchinson study is unwarranted – it is an example of a preoccupation with methods leading to misleading conclusions – as detailed information about the intervention or data on the short-term effects of the program have never been reported (see further discussion below). There is no way to judge whether or not it was a good example of the social influences approach or another watered-down approach like the DARE program.

## Reviews of Long-term effects

As noted above, Thomas and Perera concluded that there is little evidence of the long-term effectiveness of smoking prevention programs. Other recent reviews also raised questions about the long-term (high school or beyond) effects of school-based smoking prevention programs.

**Wiehe et al**. [43] conducted a meta-analysis of the only 8 studies they could find with results reported at grade 12 or age 18. These included evaluations of programs of known ineffectiveness from prior studies (e.g., Hutchinson and DARE). Other studies included in the meta-analysis were early studies of the social influences approach [47,61] that, in retrospect, we should never have expected to have long-term, or even medium-term, effects. These programs were initial small-scale experimental tests of the social influences approach that included only 5–10 sessions in one or two grades without any boosters or programming in high school. Another was Project ALERT, which consisted of only 8 sessions in 7th grade and three booster sessions in 8th grade [44]. Clearly, programs need to include more sessions, preferably with some in high school, to be effective in the long term. This is a conclusion that could have been proposed by Wiehe et al but wasn't.

Of the studies reviewed by Wiehe et al., only the Life Skills Training (LST) program, which is an interactive program of 15 sessions in 7th grade, 10 in 9th grade, and 5 in 10th grade that incorporates the social influences approach as well as other general personal and social skills, was effective at long-term follow-up. Wiehe et al. concluded that "there is little evidence to suggest that existing programs produce medium-term decreases in smoking prevalence (page 168)." In an editorial comment, Glantz and Mandel [53] misleadingly stated that the Wiehe et al. review of long-term trials "convincingly shows that they are not effective (page 157)." [62]. They then discount the LST program evaluation because of the use of one-tailed t-tests and the failure to take account of multiple comparisons. However, it is perfectly appropriate to use one-tailed t-tests when a clear hypothesis is stated, and adjusting for multiple comparisons would not have eliminated the significant effects. In addition, the short-term effects of LST have been replicated in multiple studies (see below).

Glantz and Mandel suggest that all aspects of smoking education should be integrated into regular core curriculum classes. This approach has not been shown to be effective. Furthermore, it is not likely to happen in the near future because of the current demands on schools, nor is it likely to be effective because one would expect much less adherence to the program components if the program was delivered by multiple teachers.

**Skara and Sussman **[21] reviewed studies of 25 tobacco and other drug prevention programs that included long-term follow-ups (at least 24 months). They found that 18 of the 25 studies reported significant short-term effects and 15 of 25 reported significant long-term effects. Of 17 studies with pretest and posttest data, 11 (65%) reported significant long-term effects, with an average reduction in the percentage of baseline nonusers who initiated smoking in the program condition relative to control conditions of 11.4% (range 9 to 14.2%). Of the studies with significant short-term effects, 72% (13 of 18) were found to have significant long-term effects. Results also indicated that program effects were less likely to decay for programs with extended programming or booster sessions.

## Summary of review of reviews

### Although meta-analyses and systematic reviews provide some very useful information (as well as some misleading information sometimes) for scholars, they do not provide enough information of value to policy makers and practitioners

Indeed, meta-analyses can obscure some kinds of information, particularly when there are wide variations between the types of interventions being reviewed. Meta-analyses make more sense in medicine, where the effects of the same drug or procedure can be estimated from multiple trials. In a field like school-based smoking prevention, one is often comparing different kinds of programs with differing formats, theoretical orientations, targeted behaviors, and targeted populations and age groups. Furthermore, different programs were developed by different researchers or practitioners with different theoretical or philosophical orientations (sometimes even when they claim to be the same), and implemented by different providers who, themselves, have different training and readiness for the work. There are also large differences among studies in research design and the measurement of smoking behavior. It really takes someone who is intimately familiar with a body of research and program development to conduct the most useful kind of in-depth review of a field like smoking prevention, where different programmers and programs have different training, theoretical bases and degree of understanding of children, youth and their families and other social settings, such as peer groups, schools and communities.

Despite the above short-comings, the meta-analyses by Tobler and Hwang provide clear directions on what types of programs are most effective. On the basis of a systematic review of reviews and individual studies of mediators, boosters, peer- versus adult-led, community components, Pim Cuijpers [63] developed a nice summary of the effective ingredients of effective drug prevention programs. These include:

1. Interactive delivery methods

2. The use of the social influence model (defined more broadly than by Hwang – see below)

3. Including components on norms, commitment not to use, and intentions not to use

4. Adding community components

5. Including the use of peer leaders rather than relying totally on adult providers

6. Including training and practice in the use of refusal and other life skills

In addition, meta-analyses have established that programs that have more sessions, and that continue for multiple years are more effective.

## Additional comments on reviews and meta-analyses

### Some programs are not effective

Many smoking prevention programs and activities that have received lots of attention are not effective, especially in the long term, when evaluated fully. Examples include one-time visiting speakers, other one-day special events, poster competitions, lotteries, etc. A high-profile example in the literature is the "No Smoking Class" competition, first established in Finland, where it has been carried out annually since 1989 [64], and expended to seven countries in 1997/98 [65]. Each participating class has to decide if they want to be a "Smokefree Class" for the six-month period from fall to spring. Classes monitor their (non-)smoking behavior and report it to the teacher regularly. Classes in which pupils report refraining from smoking for this period of time participate in a prize draw, where they can win a number of attractive prizes, including trips to other European countries. In addition, three lessons are provided by teachers. Nonrandomized studies with high rates of attrition of schools suggest that the program has immediate effects [64,66,67]. However, all three long-term (15-month, 18-month and 12-month) follow-up studies, two randomized trials and one not [60,64,68], demonstrated that the small immediate effects were not maintained. From theory and other research, we would expect this.

The Hutchinson project (conducted at the Fred Hutchinson Cancer Center, University of Washington) is another ineffective program, the evaluation of which has received lots of attention because it was of high quality and long term. The project was designed to be a multiyear (grades 3–10) social influences tobacco prevention program. A large randomized trial (20 school groups per condition) produced no significant effects at the end of grade 12 or 2 years later [49]. These findings are impossible to interpret, because the investigators have never reported what effects there were or were not at any other time, including prior to entering high school (when most other programs report short-term results) or at the end of the program (grade 10). *The effects of an intervention should be measured immediately or shortly after the program, and then the long-term measurement should serve to assess how permanent the effect is, or how quickly it decays*.

Certainly, one cannot use the Peterson et al. results to conclude that the social influences approach to smoking prevention is ineffective in the long-term deterrence of smoking among youth, as these authors did. These results must be interpreted in the context of the many other studies of the social influences approach in the literature [69,70].

The DARE (Drug Awareness and Resistance Education) Program was developed by the Los Angeles Police Department (LAPD) and the Los Angeles Unified School District (LAUSD) in the early 1980s. They essentially took the two variants of Project SMART (Self Management and Resistance Training) that were being tested with grade 7 students in LAUSD schools at the time [71], combined them, and added a great deal of information about drugs (including, in some variants of the program as delivered, what they looked like, where to get them, and how to take them), for LAPD police officers to deliver to grade 5 and 6 students. The results of a randomized trial of the two SMART variants found that the resistance skills program was effective and the self-management program actually led to increased drug use relative to control group students [71,72] These results, combined with our knowledge that information usually does not influence behavior very much or actually makes things worse [3,5] and the use of police officers who are not usually highly skilled teachers, make it no great surprise that DARE is not effective.

Although early nonrandomized studies suggested that DARE sometimes had small effects for elementary school students, multiple randomized trials [50,73-77] and meta-analyses [51,52] have shown that DARE has little or no impact on drug use in the short term and none in the long term. In response, DARE has developed new programs for junior and senior high school students; the junior high program also has been shown to be ineffective [78,79] and evaluations of the high school program are not yet completed.

Many health education programs are promoted as being effective even without good evaluation data. One smoking prevention program in this category is the "Tar Wars" program for elementary students operated by the American Academy of Family Physicians . Physicians or medical student volunteers go into 4^th ^and 5^th ^grade classrooms and provide one interactive 45-minute session that focuses on the short-term, image-based consequences of tobacco use. This is preceded by a lesson provided by the regular classroom teacher to teach students that, contrary to their perception, tobacco users are in the minority. The guest session is followed by a poster contest. A quasi-experimental evaluation suggests that this program produces short-term changes in knowledge [80]. Despite the lack of rigorous evaluation data, the AAFP claims that this program has reached 8 million children in 50 states and 13 countries [80], including developing countries such as Nepal (see  accessed January 18, 2008).

Another program that has been promoted as being an effective prevention program, but that had no long-term effects on smoking, is the Michigan Health Education Model. It consists of 30 lessons taught during grades 5–8, some of which include resistance skills training. Although it produced an 82% relative improvement (RI) in ever smoking at the end of the program [48,81], no significant effects on smoking behavior remained by the end of grade 12 – indeed, there was a negative effect for boys [48]. It seems that the prevention content of this program was not intensive or long enough to produce permanent effects, that additional programming might have been needed when the students were adolescents, and/or that some content may even have had a negative effect as some older informational programs did.

### Even programs of "proven effectiveness" do not always work

"Evidence-based practice" and other related terms have become common terms and standards in the U.S. and other countries in recent years. Multiple agencies have reviewed evaluation studies of substance abuse prevention programs and produced lists of "scientifically proven" or "evidence-based" programs (For one list of lists see  accessed January 18 2007). The University of Colorado provides a comparative matrix  accessed January 19, 2007). For one list of lists see  accessed January 18 2007). The University of Colorado provides a comparative matrix ( accessed January 19, 2007). The stated purpose of these lists and guides is to help decision makers, at both the federal and local levels, choose programs that are supported by the best available evidence [82]. These many lists have been confusing for practitioners because each uses different criteria and produces very different lists of "proven" programs [83,84], yet they can have a profound influence on decision making. For example, after the U.S. Department of Education compiled one such list (of 9 "exemplary" and 33 "promising" programs) with the help of a panel of eminent prevention researchers, most school districts using Federal funds believed that they had to select a program from that list [85].

Another difficulty with these lists is that some of the programs that appear on them have very limited evidence of effectiveness [84]. Gandhi and colleagues analyzed the reported effects of five prominent programs that appeared on one or more of seven prominent lists of substance abuse prevention programs. They found limited evidence showing substantial impact on drug use behavior, even at immediate posttests for most programs, with the evidence for the effectiveness of most programs coming from only one or two studies, usually conducted by the program developers. In particular, they found very few studies showing substantial impact at longer follow-ups. Thus, even many of the programs that qualify for even the most rigorous of these lists do not yet have the kinds of data to meet rigorous standards of evidence required for being of proven efficacy, effectiveness or readiness for dissemination [83].

### Policy makers and educators must be cautious about how they go about adopting and implementing smoking prevention programs, even those of "proven effectiveness"

Just because a program has been proven effective in a randomized trial does not mean that it will always be effective when delivered to different types of students (who may differ by age, culture or personality), by different providers (trained health educators, research staff, other types of visiting instructors, regular teachers), in different settings (e.g., community agencies, after-school programs). A clear example of this is the European Smoking prevention Framework Approach (ESFA) [86]. The ESFA was initiated in 1997 as a community intervention trial conducted in six countries (Denmark, Finland, the Netherlands, Portugal, Spain and the UK). It used an integrated preventive approach to smoking prevention guided by best-practice principles available at the time [12]. It targeted adolescents in and out of school as well as their parents and the schools themselves [86,87]. Short-term effects 1 year after the pre-test [87] were non-significant overall but significant effects were observed in Finland (smoking onset was 4.7% lower) and Spain (smoking onset was 3.1% lower); however, counter-productive trends were observed in Denmark and the UK. Long-term overall effects [88] were small but significant (RI = 6.4%), with larger effects in Finland (RI = 14.8% for weekly smoking, but no effect for ever smoking – see Vartiainen et al. [89] for more detail), Portugal (RI = 36% for weekly smoking, 14.9% for ever smoking) and Spain (RI = 11.8% for weekly smoking and 6.3% forever smoking), and reverse effects in the Netherlands (except for non-natives). Effects were stronger in countries where more lessons were offered, teachers were trained longer, parents and community were more engaged, and the social influence content was taught well.

The details of the ESFA programs varied considerably across countries. Such variations may have accounted for the differences in outcomes, and prior research may be informative. For example, some studies have found programs to be ineffective (or even harmful) when delivered by regular classroom teachers [90,91]. The use of peer leaders has been found to improve effectiveness [63,92], but they were not used in the ESFA. Some studies have reported effectiveness only when programs were delivered with high fidelity [91,93-96].

Programs with replicated findings are more likely than programs for which there is no prior evaluation to be effective in different settings, but only if those replications showed effectiveness in many different types of students, providers and settings in evaluations by different investigators. The program with the most replications is Life Skills Training (to be reviewed below), but most of these have been conducted by the program's developer in the U.S, with replications in Spain and Europe.

Gandhi et al [84] found that only two of the programs on the lists they reviewed had studies of long-term follow-up, [93,97]. These will be reviewed below.

### Cultural sensitivity

Cultural sensitivity is believed to be important in public health [98] and for effective prevention [99-113]. Many studies have evaluated the effectiveness of untargeted or targeted prevention curricula in white, minority, or diverse samples, but few studies have directly compared culturally relevant smoking prevention curricula with curricula that do not address cultural issues [114]. Botvin et al [115] have found that culturally targeted and non-targeted versions of their Life Skills Program were both more effective than a control condition in preventing smoking among African American and Hispanic adolescents. Another group [102,116-119] compared prevention curricula targeted to the values of several cultural groups: a Mexican American curriculum, a Black/White curriculum, and a multicultural curriculum. All three curricula were more effective than a control condition, with the Mexican American and multicultural curricula affecting more outcome variables (regardless of the students' ethnic characteristics) than the Black/White curriculum.

In a study in ethnically diverse schools (Hispanic, Asian-American, Caucasian) in Southern California, Johnson and colleagues [114,120] compared two 8-session, social-influence based curricula. One was an individualist-framed program, a version of the SMART program [72] with highly individualized content that emphasized "looking after yourself" (Project CHIPS – Choosing Healthy Influences for a Positive Self). The other was a collectivist-framed program that included cultural values from Hispanic and Asian cultures that emphasized collectivist objectives, interdependence of family members, respect for ancestors, and harmonious interpersonal relations (Project FLAVOR, Fun Learning about Vitality, Origins and Respect). They found that only the culturally sensitive curriculum (Project FLAVOR) significantly effected smoking initiation. The effects were larger for schools with large proportions of Hispanic students, and especially among the Hispanic students within those schools. Indeed, the multicultural program was effective only for Hispanic students in mostly-Hispanic schools. In contrast, the individualist-framed curriculum was effective only for Asian students in Asian/multicultural schools.

The kinds of results reported above suggest great caution is needed when implementing programs with different ethnic groups or in different cultures. On the one hand, some programs seem to be equally effective with many different groups but, on the other hand, some studies suggest that making programs culturally relevant might be very important. Clearly, we need more research on this issue. In the mean time, *any community or country adopting a program will need to evaluate it rigorously to determine its effectiveness in the new setting or culture*.

## Conclusion from review of reviews

In summary, findings from various reviews and meta-analyses suggest that school-based smoking prevention programs can have significant long-term effects if they:

(1) are interactive social influences or social skills programs that

(2) involve 15 or more sessions, including some up to at least ninth grade, that

*(3) produce substantial short-term effects*.

However, it is not easy to adopt and adapt a program for use in contexts different from those in which it was tested, especially in other cultures and countries, and great care must be taken to implement with integrity and monitor implementation and outcomes. These findings also suggest that many more programs that have reported short-term effects might also have medium- and long-term effects if they were evaluated. Unfortunately, long-term studies are relatively rare, mostly due to lack of funding. In the accompanying paper I provide a review of selected school-based smoking prevention programs that have the promise of long-term effectiveness.

## Competing interests

The author declares that they have no competing interests.

## References

[B1] Thompson EL (1978). Smoking education programs 1960–1976. American Journal of Public Health.

[B2] Beattie A (1984). Health education and the science teacher: Incitation to a debate. Education and Health.

[B3] Goodstadt MS (1978). Alcohol and drug education: Models and outcomes. Health Education Monographs.

[B4] Hwang MS, Yeagley KL, Petosa R (2004). A Meta-Analysis of Adolescent Psychosocial Smoking Prevention Programs Published Between 1978 and 1997 in the United States. Health Education & Behavior.

[B5] Goodstadt MS (1980). Drug education: A turn on or a turn off. Journal of Drug Education.

[B6] Petrosino A, Turpin-Petrosino C, Finckenauer JO (2000). Well-Meaning Programs Can Have Harmful Effects! Lessons From Experiments of Programs Such as Scared Straight. Crime & Delinquency.

[B7] Evans RI (1976). Developing a social psychological strategy of deterrence. Preventive Medicine.

[B8] McGuire WJ, Berkowitz L (1964). Inducing resistance to persuasion. Advances in Experimental Social Psychology.

[B9] DHHS (1991). Strategies to Control Tobacco Use in the United States: A Blueprint for Public Health Action in the 1990's.

[B10] DHHS (1994). Preventing tobacco use among young people: A report of the Surgeon General. Atlanta, Georgia: Public Health Service, Centers for Disease Control and Prevention, Office on Smoking and Health.

[B11] Lynch BJ, Bonnie RJ, editors (1994). Growing Up Tobacco Free.

[B12] Glynn TJ (1989). Essential elements of school-based smoking prevention programs. Journal of School Health.

[B13] Bonnie RJ, Stratton KR, Wallace RB, editors (2007). Ending the Tobacco Problem: A Blueprint for the Nation.

[B14] Burns DM (1992). Positive evidence on effectiveness of selected smoking prevention programs in the United States. Journal of the National Cancer InstituteMonographs.

[B15] DHHS (2000). Reducing tobacco use: A report of the Surgeon General.

[B16] Hansen WB (1992). School-based substance use prevention: A review of the state of the art in curriculum, 1980–1990. Health Education Research: Theory and Practice.

[B17] Krowchuk HV (2005). Effectiveness of adolescent smoking prevention strategies. MCN Am J Matern Child Nurs.

[B18] Lantz PM, Jacobson PD, Warner KE, Wasserman J, Pollack HA, Berson J (2000). Investing in youth tobacco control: a review of smoking prevention and control strategies. Tobacco Control.

[B19] Lober Aquilino M, Lowe JB (2004). Approaches to tobacco control: the evidence base. European Journal Of Dental Education.

[B20] Pentz MA (1999). Effective prevention programs for tobacco use. Nicotine & Tobacco Research.

[B21] Skara S, Sussman S (2003). A review of 25 long-term adolescent tobacco and other drug use prevention program evaluations. Preventive Medicine.

[B22] Vickers KS, Thomas JL, Patten CA, Mrazek DA (2002). Prevention of tobacco use in adolescents: review of current findings and implications for healthcare providers. Curr Opin Pediatr.

[B23] Sussman S, Lichtman K, Ritt A, Pallonen U (1999). Effects of thirty four adolescent tobacco use cessation and prevention trials on regular users of tobacco products. Subst Use Misuse.

[B24] Ranney LM, Melvin C, Lux L, McClain E, Morgan L, Lohr KN (2006). Tobacco Use: Prevention, Cessation, and Control. Research Triangle, NC: R. T. I.

[B25] Glynn TJ, Manley MW, Gerlach KK, Shopland DR (1996). Public health approaches to tobacco use prevention and cessation in the US. J Public Health Manag Pract.

[B26] Burns D, Amara R, Ruppert W (2002). Changing Adolescent Smoking Prevalence. Where it is and why Smoking and Tobacco Control: National Cancer Institute.

[B27] Warner KE, Jacobson PD, Kaufman NJ (2003). Innovative approaches to youth tobacco control: introduction and overview.

[B28] Dobbins M, DeCorby K, Manske S, Goldblatt E (2008). Effective practices for school-based tobacco use prevention. Preventive Medicine.

[B29] Backinger CL, Fagan P, Matthews E, Grana R (2003). Adolescent and young adult tobacco prevention and cessation: current status and future directions. British Medical Journal.

[B30] Buttross LS, Kastner JW (2003). A brief review of adolescents and tobacco: what we know and don't know. Am J Med Sci.

[B31] Stead M, Hastings G, Tudor-Smith C (1996). Preventing Adolescent smoking: a review of options. Health Education Journal.

[B32] Sussman S (2001). School-based tobacco use prevention and cessation: where are we going. Am J Health Behav.

[B33] Thomas R, Perera R (2007). School-based programmes for preventing smoking (Review). The Cochrane Library.

[B34] Tingle LR, DeSimone M, Covington B (2003). A meta-evaluation of 11 school-based smoking prevention programs. Journal of School Health.

[B35] Torre G, Chiaradia G, Ricciardi G (2005). School-based smoking prevention in children and adolescents: review of the scientific literature. Journal of Public Health.

[B36] Park E (2006). School-based smoking prevention programs for adolescents in South Korea: a systematic review. Health Education Research.

[B37] Flay BR, Bonnie RJ, Stratton K, Wallace RB (2007). The Long-Term Promise of Effective School-Based Smoking Prevention Programs. Ending the tobacco problem: A blueprint for the nation.

[B38] Black DR, Tobler NS, Sciacca JP (1998). Peer helping/involvement: An efficacious way to meet the challenge of reducing alcohol, tobacco, and other drug use among youth?. Journal of School Health.

[B39] Bruvold WH (1993). A meta-analysis of adolescent smoking prevention programs. American Journal of Public Health.

[B40] Rooney BL, Murray DM (1996). A Meta-Analysis of Smoking Prevention Programs After Adjustment for Errors in the Unit of Analysis. Health Education & Behavior.

[B41] Tobler NS, Roona MR, Ochshorn P, Marshall DG, Streke AV, Stackpole KM (2000). School-based adolescent drug prevention programs: 1998 meta-analysis. The Journal of Primary Prevention.

[B42] Tobler NS, Stratton HH (1997). Effectiveness of school-based drug prevention programs: A meta-analysis of the research. Journal of Primary Prevention.

[B43] Wiehe SE, Garrison MM, Christakis DA, Ebel BE, Rivara FP (2005). A systematic review of school-based smoking prevention trials with long-term follow-up. Journal of Adolescent Health.

[B44] Ellickson PL, Bell RM, McGuigan K (1993). Preventing adolescent drug use: Long-term results of a junior high program. American Journal of Public Health.

[B45] Hawkins DJ, Catalano RF, Kosterman R, Abbott R, Hill K (1999). Preventing adolescent health-risk behaviors by strengthening protection during childhood. Arch Pediatr Adolesc Med.

[B46] Murray DM, Pirie P, Luepker RV, Pallonen U (1989). Five-and six-year follow-up results from four seventh-grade smoking prevention strategies. Journal of Behavioral Medicine.

[B47] Shean RE, de Klerk NH, Armstrong BK, Walker NR (1994). Seven-year follow-up of a smoking-prevention program for children. Aust J Public Health.

[B48] Shope JT, Copeland LA, Kamp ME, Lang SW (1998). Twelfth grade follow-up of the effectiveness of a middle school-based substance abuse prevention program. Journal of Drug Education.

[B49] Peterson AV, Kealey KA, Mann SL, Marek PM (2000). Hutchinson Smoking Prevention Project: Long-term randomized trial in school-based tobacco use prevention – results on smoking. Journal of the National Cancer Institute.

[B50] Ennett ST, Rosenbaum DP, Flewelling RL, Bieler GS, Ringwalt CL, Bailey SL (1994). Long-Term Evaluation of Drug Abuse Resistance Education. Addictive Behaviors.

[B51] Ennett ST, Tobler NS, Ringwalt CL, Flewelling RL (1994). How Effective Is Drug Abuse Resistance Education? A Meta-Analysis of Project DARE Outcome Evaluations. American Journal of Public Health.

[B52] West SL, O'Neal KK (2004). Project D.A.R.E. outcome effectiveness revisited. American Journal of Public Health.

[B53] Glantz SA, Mandel LL (2005). Since school-based tobacco prevention programs do not work, what should we do?. Journal of Adolescent Health.

[B54] Flay BR (2009). School-based smoking prevention programs with the promise of long-term effectiveness. Tobacco Induc Dis.

[B55] Tobler NS (2000). Lessons Learned. The Journal of Primary Prevention.

[B56] Flay BR (2000). Approaches to substance use prevention utilizing school curriculum plus social environment change. Addictive Behaviors.

[B57] Siddiqui O, Flay BR, Hu FB (1996). Factors affecting attrition in a longitudinal smoking prevention study. Preventive Medicine.

[B58] Lipsey MW, Crosse S, Dunkle J, Pollard J, Stobart G, Cordray DS (1985). Evaluation: The state of the art and the sorry state of science.

[B59] Ausems M, Mesters I, van Breukelen G, De Vries H (2004). Effects of in-school and tailored out-of-school smoking prevention among Dutch vocational school students. Health Education Research.

[B60] Crone MR, Reijneveld SA, Willemsen MC, van Leerdam FJ, Spruijt RD, Sing RA (2003). Prevention of smoking in adolescents with lower education: A school based intervention study. Journal of Epidemiology and Community Health.

[B61] Flay BR (1989). Six-year follow-up of the first Waterloo school smoking prevention trial. American Journal of Public Health.

[B62] Flay BR (2005). School-based smoking prevention research: Letter to the editor. Journal of Adolescent Health.

[B63] Cuijpers P (2002). Effective ingredients of school-based drug prevention programs A systematic review. Addictive Behaviors.

[B64] Vartiainen E, Saukko A, Paavola M, Vertio H (1996). 'No Smoking Class' competitions in Finland: their value in delaying the onset of smoking in adolescence. Health Promotion International.

[B65] Hanewinkel R, Wiborg G, Paavola M, Vartiainen E (1998). European smoke-free class competition. Tobacco Control.

[B66] Hanewinkel R, Wiborg G (2002). Primär-und Sekundärprävention des Rauchens im Jugendalter: Effekte der Kampagne „Be Smart-Don't Start" Primary and Secondary Prevention of Smoking in Adolescents: Results of the Campaign „Be Smart-Don't Start". Gesundheitswesen.

[B67] Wiborg G, Hanewinkel R (2002). Effectiveness of the "Smoke-Free Class Competition" in Delaying the Onset of Smoking in Adolescence. Preventive Medicine.

[B68] Schulze A, Mons U, Edler L, Pötschke-Langer M (2006). Lack of sustainable prevention effect of the "Smoke-Free Class Competition" on German pupils. Preventive Medicine.

[B69] Botvin GJ, Sussman S, Biglan A (2001). EDITORIAL: The Hutchinson Smoking Prevention Project: A Lesson on Inaccurate Media Coverage and the Importance of Prevention Advocacy. Prevention Science.

[B70] Sussman S, Hansen WB, Flay BR, Botvin GJ (2001). Re: Hutchinson Smoking Prevention Project: Long-term randomized trial in school-based tobacco use prevention – results on smoking. Journal of the National Cancer Institute.

[B71] Graham JW, Johnson CA, Hansen WB, Flay BR, Gee M (1990). Drug use prevention programs, gender, and ethnicity: Evaluation of three seventh-grade project SMART cohorts. Preventive Medicine.

[B72] Hansen WB, Johnson CA, Flay BR, Graham JW, Sobel JL (1988). Affective and social influences approaches to the prevention of multiple substance abuse among seventh grade students: Results from Project SMART. Preventive Medicine.

[B73] Clayton RR, Cattarello AM, Johnstone BM (1996). The effectiveness of Drug Abuse Resistance Education (Project DARE): 5-Year follow-up results. Preventive Medicine.

[B74] Dukes RL, Ullman JB, Stein JA (1996). Three-Year Follow-Up of Drug Abuse Resistance Education (D.A.R.E.). Evaluation Review.

[B75] Lynam D, Milich R, Zimmerman R, Novak SP, Logan TK, Martin C (1999). Project DARE: No Effects at 10-Year Follow-Up. Journal of Consulting and Clinical Psychology.

[B76] Rosenbaum D, Flewelling RL, Bailey SL, Ringwalt CL, Wilkinson DL (1994). Cops in the classroom: A longitudinal evaluation of Drug Abuse Resistance Education (DARE). Journal of Research in Crime and Deinquency.

[B77] Rosenbaum DP, Hanson GS (1998). Assessing the effects of school-based drug education: A six-year multilevel analysis of Project D.A.R.E. Journal of Research in Crime and Delinquency.

[B78] Perry CL, Komro KA, Veblen-Mortenson S, Bosma LM, Farbakhsh K, Munson KA (2003). A Randomized Controlled Trial of the Middle and Junior High School DARE and DARE Plus Programs. Archives of Pediatrics and Adolescent Medicine.

[B79] Perry CL, Komro KA, Veblen-Mortenson S (2000). The Minnesota DARE PLUS Project: Creating community partnerships to prevent drug use and violence. Journal of School Health.

[B80] Cain JJ, Dickinson WP, Fernald D, Bublitz C, Dickinson LM, West D (2006). Family physicians and youth tobacco-free education: Outcomes of the Colorado Tar Wars program. Journal of the American Board of Family Medicine.

[B81] Shope JT, Copeland LA, Marcoux BC, Kamp ME (1996). Effectiveness of a school-based substance abuse prevention program. Journal of Drug Education.

[B82] Petrosino A (2003). Standards for Evidence and Evidence for Standards: The Case of School-Based Drug Prevention. The ANNALS of the American Academy of Political and Social Science.

[B83] Flay BR, Biglan A, Boruch RF, Castro FG, Gottfredson D, Kellam S (2005). Standards of evidence: Criteria for efficacy, effectiveness, and dissemination. Prevention Science.

[B84] Gandhi AG, Murphy-Graham E, Petrosino A, Chrismer SS, Weiss CH (2007). The Devil Is in the Details: Examining the Evidence for" Proven" School-Based Drug Abuse Prevention Programs. Evaluation Review.

[B85] Weiss CH, Murphy-Graham E, Birkeland S (2005). An Alternate Route to Policy Influence: How Evaluations Affect DARE. American Journal of Evaluation.

[B86] De Vries H, Mudde A, Leijs I, Charlton A, Vartiainen E, Buijs G (2003). The European Smoking prevention Framework Approach (EFSA): an example of integral prevention. Health Education Research.

[B87] De Vries H, Mudde A, Kremers S, Wetzels J, Uiters E, Ariza C (2003). The European Smoking Prevention Framework Approach (ESFA): short-term effects. Health Education Research.

[B88] De Vries H, Dijk F, Wetzels J, Mudde A, Kremers S, Ariza C (2006). The European Smoking prevention Framework Approach (ESFA): effects after 24 and 30 months.

[B89] Vartiainen E, Pennanen M, Haukkala A, Dijk F, Lehtovuori R, De Vries H (2007). The effects of a three-year smoking prevention programme in secondary schools in Helsinki. The European Journal of Public Health.

[B90] Bell RM, Ellickson PL, Harrison ER (1993). Do drug prevention effects persist into high school? How Project ALERT did with ninth graders. Preventive Medicine.

[B91] Botvin GJ, Baker E, Filazzola AD, Botvin EM (1990). A cognitive-behavioral approach to substance abuse prevention: one-year follow-up. Addictive behaviors.

[B92] Klepp K-I, Halper A, Perry CL (1986). The efficacy of peer leaders in drug abuse prevention. Journal of School Health.

[B93] Botvin GJ, Baker E, Dusenbury L, Botvin EM, Diaz T (1995). Long-term follow-up results of a randomized drug abuse prevention trial in a white middle-class population. JAMA.

[B94] Botvin GJ, Baker E, Dusenbury L, Tortu S, Botvin EM (1990). Preventing adolescent drug abuse through a multimodal cognitive-behavioral approach: Results of a 3-year study. Journal of Consulting and Clinical Psychology.

[B95] Botvin GJ, Dusenbury L, Baker E, James-Ortiz S, Kerner J (1989). A skills training approach to smoking prevention among hispanic youth. Journal of Behavioral Medicine.

[B96] Pentz MA, Trebow EA, Hansen WB, MacKinnon DP, Dwyer JH, Johnson CA (1990). Effects of program implementation on adolescent drug use behavior: The Midwestern Prevention Project (MPP). Evaluation Review.

[B97] Pentz MA, MacKinnon DP, Flay BR, Hansen WB, Johnson CA, Dwyer JH (1989). Primary prevention of chronic diseases in adolescence: Effects of the Midwestern Prevention Project on tobacco use. American Journal of Epidemiology.

[B98] Resnicow K, Baranowski T, Ahluwalia JS, Braithwaite RL (1999). Cultural sensitivity in public health: Defined and demystified. Ethnicity & Disease.

[B99] Chen MS (2004). Challenges in tobacco use prevention among minority youth. Cancer Epidemiol Biomarkers Prev.

[B100] Ferketich AK (2007). Design and evaluation of a tobacco-prevention program targeting Chinese American youth in New York City. Nicotine & Tobacco Research.

[B101] Flay BR, Graumlich S, Segawa E, Burns JL, Holliday MY, Aban-Aya I (2004). Effects of 2 prevention programs on high-risk behaviors among African American youth: A randomized trial. Archives of Pediatric Adolescent Medicine.

[B102] Hecht ML, Krieger JLR (2006). The Principle of Cultural Grounding in School-Based Substance Abuse Prevention: The Drug Resistance Strategies Project. Journal of Language and Social Psychology.

[B103] Klonoff EA, Landrine H (1999). Acculturation and Cigarette Smoking Among African Americans: Replication and Implications for Prevention and Cessation Programs. Journal of Behavioral Medicine.

[B104] LaFromboise T, Coleman HLK, Gerton J (1993). Psychological impact of biculturalism: Evidence and theory. Psychological Bulletin.

[B105] Litrownik AJ, Elder JP, Campbell NR, Ayala GX, Slymen DJ, Parra-Medina D (2000). Evaluation of a Tobacco and Alcohol Use Prevention Program for Hispanic Migrant Adolescents: Promoting the Protective Factor of Parent-Child Communication. Preventive Medicine.

[B106] Lynagh M (1997). School health promotion programs over the past decade: a review of the smoking, alcohol and solar protection literature. Health Promotion International.

[B107] Miranda J, Bernal G, Lau A, Kohn L, Hwang WC, LaFromboise T (2005). State of the science on psychological intervention for ethnic minorities. Annual Review of Clinical Psychology(2005).

[B108] Schinke SP, Botvin GJ, Orlandi MA (1990). African-American and Hispanic-American adolescents, HIV infection and preventive intervention. AIDS Education and Prevention.

[B109] Schinke SP, Gilchrist LD, Schilling RF, Walker R, Dale (1987). Preventing substance abuse among American Indian and Alaska Native youth: Research issues and strategies. Journal of Social Service Research.

[B110] Schinke SP, Orlandi MA, Botvin GJ, Gilchrist LD, Trimble JE, Locklear VS (1988). Preventing substance abuse among American-Indian adolescents: A bicultural competence skills approach. Journal of Counseling Psychology.

[B111] Shelley D, Fahs M, Scheinmann R, Swain S, Qu J, Burton D (2004). Acculturation and Tobacco Use Among Chinese Americans. Am Public Health Assoc.

[B112] Sussman S, Yang D, Baezconde-Garbanati L, Dent CW (2003). Drug Abuse Prevention Program Development: Results among Latino and Non-Latino White Adolescents. Evaluation & the Health Professions.

[B113] Velez MB (2001). The Role of Public Social Control in Urban Neighborhoods: A Multi-Level Analysis of Victimization. Criminology.

[B114] Johnson CA, Unger JB, Ritt-Olson A, Palmer PH, Cen SY, Gallaher P (2005). Smoking prevention for ethnically diverse adolescents: 2-year outcomes of a multicultural, school-based smoking prevention curriculum in Southern California. Preventive Medicine.

[B115] Botvin GJ, Schinke SP, Epstein JA (1995). Effectiveness of Culturally Focused and Generic Skills Training Approaches to Alcohol and Drug Abuse Prevention Among Minority Adolescents: Two-Year Follow-Up Results. Psychology of Addictive Behaviors.

[B116] Gosin M, Marsiglia FF, Hecht ML (2003). keepin'it REAL: A drug resistance curriculum tailored to the strengths and needs of pre-adolescents of the southwest. Journal of Drug Education.

[B117] Hecht ML, Graham JW, Elek E (2006). The Drug Resistance Strategies Intervention: Program Effects on Substance Use. Health Communication.

[B118] Hecht ML, Marsiglia FF, Elek E, Wagstaff DA, Kulis S, Dustman P (2003). Culturally Grounded Substance Use Prevention: An Evaluation of the keepin'it REAL Curriculum. Prevention Science.

[B119] Warren JR, Hecht ML, Wagstaff DA, Elek E, Ndiaye K, Dustman P (2006). Communicating Prevention: The Effects of the keepin'it REAL Classroom Videotapes and Televised PSAs on Middle-School Students' Substance Use. Journal of Applied Communication Research.

[B120] Johnson CA, Cen S, Gallaher P, Palmer PH, Xiao L, Ritt-Olson A (2007). Why Smoking Prevention Programs Sometimes Fail: Does Effectiveness Depend on Sociocultural Context and Individual Characteristics?. Cancer Epidemiology Biomarkers & Prevention.

